# Oviposition activity of *Haemagogus leucocelaenus* (Diptera: Culicidae) during the rainy and dry seasons, in areas with yellow fever virus circulation in the Atlantic Forest, Rio de Janeiro, Brazil

**DOI:** 10.1371/journal.pone.0261283

**Published:** 2021-12-13

**Authors:** Jeronimo Alencar, Cecilia Ferreira de Mello, Paulo José Leite, Amanda Queiroz Bastos, Shayenne Olsson Freitas Silva, Michele Serdeiro, Júlia dos Santos Silva, Gerson Azulim Müller

**Affiliations:** 1 Diptera Laboratory, Oswaldo Cruz Institute (FIOCRUZ), Rio de Janeiro, Brazil; 2 Graduate Program in Animal Biology, Institute of Biology, Federal Rural University of Rio de Janeiro, Rio de Janeiro, Brazil; 3 Graduate Program in Tropical Medicine, Oswaldo Cruz Institute (Fiocruz), Rio de Janeiro, Brazil; 4 Farroupilha Federal Institute of Education Science and Technology, Panambi, Rio Grande do Sul, Brazil; University of Texas Medical Branch, UNITED STATES

## Abstract

The present study aims to analyze the effectiveness of ovitraps in the capture of *Hg leucocelaenus* eggs and evaluate the influence of the dry and rainy seasons on their abundance and hatching rates. The eggs were collected in the Atlantic Forest of State of Rio de Janeiro, Brazil, an area in which the yellow fever virus is known to circulate. We distributed 15 ovitraps in three sampling points, with five ovitraps per point. We distributed 15 ovitraps in three sampling points on trees within a forested area, which were sequentially numbered, monitored, and replaced every two weeks from October 2016 to April 2018. There was a high dominance of *Hg*. *leucocelaenus* eggs (98.4%) and a variation in egg hatching rates between the wet and dry seasons. These rates were 1.5 times higher in the rainy season than in the dry season. The rainy season also showed a greater abundance of eggs and higher values of ovitrap positivity and egg density indexes in the installed ovitraps. The abundances of *Hg*. *leucocelaenus* eggs were positively correlated with mean monthly temperature and air humidity but not significantly correlated with accumulated precipitation. These results, as well as their implications for the possible use of ovitraps to monitor vector mosquitoes of yellow fever in the study region, are discussed.

## Introduction

*Haemagogus leucocelaenus* (Dyar and Shannon, 1924) is a mosquito species with a geographic distribution that extends from Argentina to Trinidad [1, 2]. It is commonly found in Brazil, especially in the central-western, southeastern, and southern regions [3]. This species is epidemiologically important since it is considered one of the main vectors of the yellow fever virus (YFV) in forest areas, along with *Haemagogus janthinomys* Dyar (1921), the primary vector of this virus [4]. Recently, the Zika virus was detected from *Hg*. *leucocelaenus*, suggesting that it may also be involved in the transmission cycles of other arboviruses [5].

According to Alencar et al. [6, 7], *Hg*. *leucocelaenus* is eclectic in terms of feeding habits and oviposition patterns. These mosquitoes lay their eggs in hollow trees or bamboo internodes in the canopy and near the forest floor. The breeding sites used by *Hg*. *leucocelaenus* are affected by water level fluctuations, with potential breeding areas running out of water in drier and hotter periods. Therefore, eggs from this species are laid on moist plant substrates near the water surface where they need to come into contact with water repeatedly in order to hatch [8, 9].

Schaeffer et al. [10] studied two species of *Aedes* that breed in tree holes and found that precipitation is the primary limiting factor for egg hatching and larval development in such breeding sites. Campos and Sy [11] observed that precipitation and temperature strongly influence the hatching rate of *Ochlerotatus albifasciatus* (Macquart, 1838) eggs. Climate change can affect biodiversity at different levels by accelerating some individuals’ metabolism and affecting the food chains and ecological interactions of populations and communities [12]. According to Alencar et al. [13], the activity level of different mosquito species is directly influenced by climatic variables, such as temperature and air humidity. Since there are not just some vector species affected by these abiotic factors but entire populations of many Culicidae species and other arthropods as well. However, while climate has been shown to influence the abundance of vector mosquitoes, little is known about the effects of climatic variations on the dynamics of oviposition and the hatching rates of eggs, particularly those of *Hg*. *leucocelaenus*.

Population monitoring of *Hg*. *leucocelaenus* in the nature, especially in areas with the occurrence of YFV, is performed invariably through the collection of adults using entomological nets, manual vacuums, and CDC traps [14, 15]. However, collecting immature forms of these mosquitoes is challenging since it is not always possible to locate holes in trees in the forest. In addition, when breeding sites are located, the visualization of eggs is very difficult, hampering the adoption of this practice by monitoring programs of YFV vectors in Brazil. Ovitraps are traps that simulate potential breeding sites for mosquitoes, basically consisting of a black plastic pot, water, and a wooden straw. They are widely used in *Aedes aegypti* monitoring programs of Brazilian urban areas [16]. However, this trap also seems to work well for some groups of mosquitoes that occur in wild areas, especially those that use phytotelmata breeding sites, such as mosquitoes of the genera *Aedes*, *Haemagogus*, *Limatus*, and *Toxorhynchites* [17, 18].

The present study aimed to analyze the effectiveness of ovitraps for capturing *Hg leucocelaenus* eggs, as well as evaluate the influence of the dry and rainy seasons on the abundance and hatching rates of this mosquito species.

## Materials and methods

### Ethics statement

The permanent license for collecting, capturing, and transporting biological material was granted by the Biodiversity Authorization and Information System (SISBIO)/ Chico Mendes Institute for Biodiversity Conservation (ICMBio) under the number 34911–1. All team members were previously vaccinated against YF.

### Study area

The Sana Environmental Protection Area (SPA) is a 15.7 ha reserve located in an area of secondary Atlantic Forest in the municipality of Macaé, State of Rio de Janeiro, Brazil. This SPA is situated in a valley surrounded by mountains and consists of a large area of dense ombrophilous vegetation, including several waterfalls and a high diversity of fauna and flora. The Sana River basin, present in most of the SPA territory, is the largest and most important water source of the Macaé River [19].

**Fig 1:**
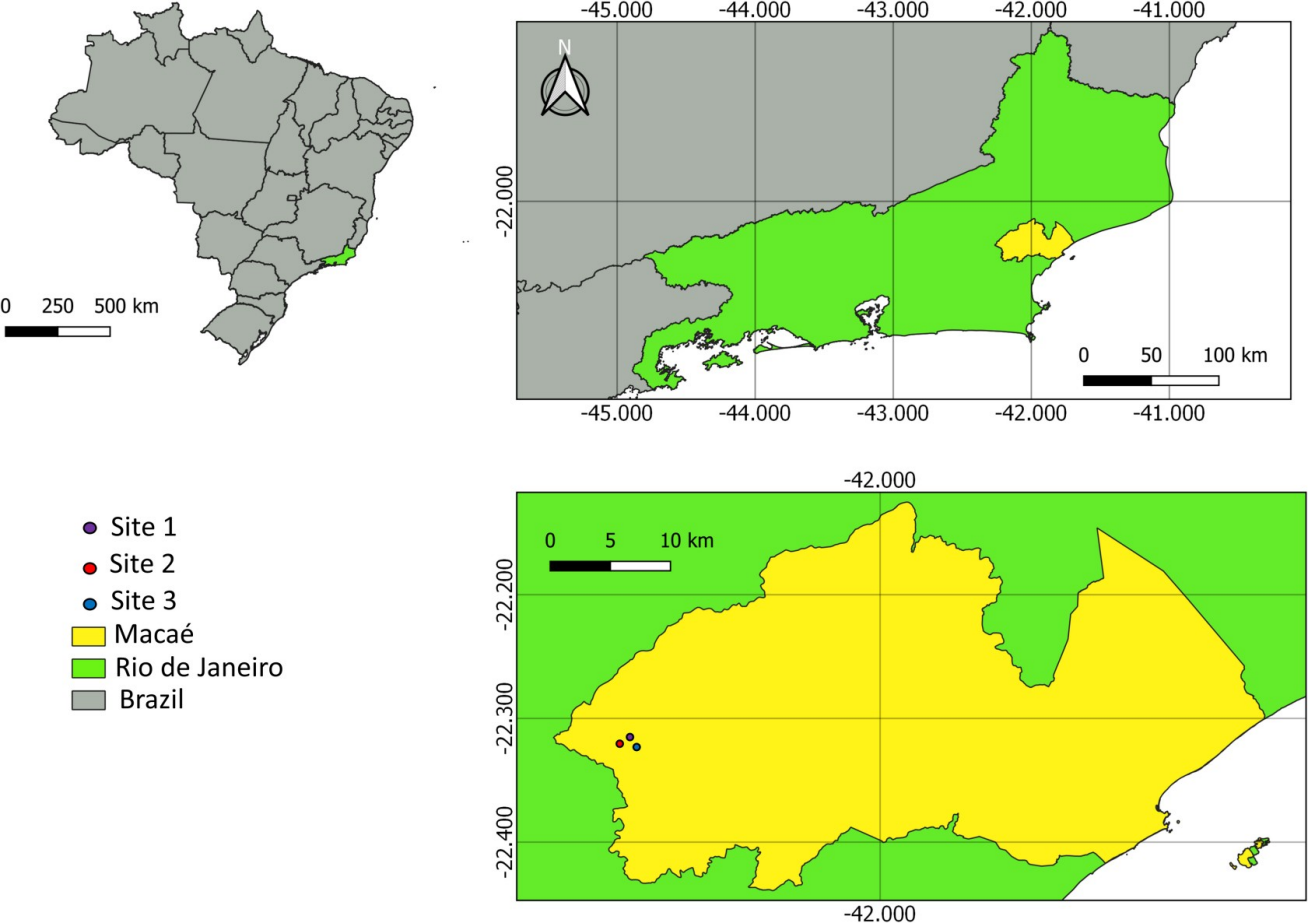
Sampling sites in the Sana Environmental Protection Area, located in the sixth district of Macaé, Rio de Janeiro, Brazil. Maps were prepared in QGIS 3.14.16software and edited in Adobe Photoshop CS5 and CorelDraw X5. Reprinted from QGIS 3.14.16, a program under a CC BY license, with permission from Jeronimo Alencar—Fiocruz, original copyright 2021.

The sampling sites and their characteristics were as follows: site 1, with a dense shrub layer and tall trees very close to each other (22°20’01.3’’S, 42°12’24.0’’W); site 2, located on the banks of the Sana River, with a mosquito fauna influenced by bamboo vegetation (22°20’02.9’’S, 42°12’28.3’’W); and site 3, with vegetation cover similar to site 1 (22°20’02.9’’S, 42°12’31.1’’W). Maps were prepared in ArcGIS 10 software and edited in Adobe Photoshop CS5 and CorelDraw X5. The sampling locations are shown in (Fig 1).

The state of Rio de Janeiro is characterized by having a hot and rainy climate between November and April (rainy period) and a cold and dry climate between May and October (dry period) [20].

### Collections and laboratory procedures

We used ovitraps to collect eggs, which consisted of 500 mL black containers without a lid, resembling a plant vase. Inside, the ovitraps contained four wooden oviposition paddles (2.5 cm × 14 cm), vertically held in place with a clip and textured surfaces to facilitate oviposition. We added 300 ml of natural water from water bodies close to the collection sites and approximately 100g of leaf litter in each ovitrap in an effort to recreate a microecosystem resembling the natural environment (S1 Fig). We distributed 15 ovitraps in three sampling points on trees within a forested area, which were sequentially numbered, monitored, and replaced every two weeks from October 2016 to April 2018 [21]. Ovitraps were installed in trees at a height of 2.50 meters from the ground in the forest, with nylon ropes and wire to hold the ovitraps to the trees [22, 23]. After the ovitraps were collected, they were packed in a polyethylene box and sent to the Diptera Laboratory of the Oswaldo Cruz Institute in the city of Rio de Janeiro. Positive paddles (containing eggs) were separated in the laboratory, where the eggs were counted and immersed in transparent trays containing dechlorinated water. The eggs were then placed in a controlled experimental environment in a laboratory greenhouse with a thermoperiod regulated at a temperature of 28°C ± 1°C, relative air humidity of 75–90%, and a 12 h day/12 h night cycle. After three days, the paddles were removed from the water and left to dry in the air for another three days to quantify the hatched larvae. Immature forms were reared as described by Alencar [6]. Positive paddles were immersed as many times as needed to allow hatching of all eggs present. Thus, the eggs were subjected to repeated cycles of immersions and drying until all of them had hatched. These conditions allowed us to keep the specimens alive until adulthood for specific identification following the methodology described by Arnell [24].

Adult identification was carried out at the species level by direct observation of morphological characters using a stereomicroscope. We consulted the respective specific descriptions/diagnoses of dichotomous keys elaborated by Arnell [24], and Forattini [25]. Following species identification, all specimens were deposited in the Entomological Collection of the Oswaldo Cruz Institute, Fiocruz, Rio de Janeiro, under the “Sana Environmental Protection Area Collection” designation. The abbreviations for mosquito genera adopted are those proposed by Reinert [26].

### Data analysis

After quantifying the collected eggs, we calculated the ovitrap positivity index (OPI = No. of positive traps/No. of examined traps x 100%) and egg density index (EDI = No. of eggs/No. of positive traps) [27].

We first obtained descriptive statistics, including absolute and relative frequencies, and then performed the Shapiro–Wilk test to verify the normality of the data. Subsequently, we used Welch’s modified 2-sample t-test, which does not assume equal variances between samples, to test for differences in the abundance of eggs collected between the dry and rainy periods. The number of hatches (fertility) was compared to the total number of eggs collected in the dry and rainy periods using Yates’s chi-squared test, estimates of odds ratios, and 95% confidence intervals. The Spearman’s rank correlation coefficient was used to analyze the correlation between eggs abundance and monthly mean temperature, monthly mean relative air humidity, and monthly accumulated precipitation. We conducted the statistical analysis in GraphPad Prism version 8.01 for Windows (San Diego, California, USA), with the significance level of p < 0.05.

The average values of temperature and relative humidity of the air and accumulated precipitation were obtained from the period of 30 days prior to the day of each collection from daily readings of climatic conditions. These climatic parameters were obtained from Brazil’s Institute of Meteorology (INMET). Furthermore, the beginning, end, and duration of each period were determined using the criterion proposed by Marcuzzo et al. [28], which was based on a series of data spanning 30 years.

## Results

We collected 13,419 mosquito eggs, of which 11,129 hatched and were identified as *Hg*. *leucocelaenus* (n = 10,946; 98.4%), *Ae*. *terrens* (Walker, 1856) (n = 172; 1.5%), and *Hg*. *janthinomys* (n = 11; 0.1%). A total of 2,290 eggs did not hatch and consequently could not be identified (S1 Table).

Of all the eggs collected during the rainy season, 83.5% hatched and 16.5% did not hatch. Meanwhile, 77.6% of the eggs collected during the dry period hatched, whereas 22.4% did not (Table 1). The chi-square test showed that the hatching rate was significantly higher during the rainy season (Yates corrected x2 = 30.60, df = 1, p < 0.0001). The odds ratio was 1.5 (CI 95%, 1.281 to 1.676), indicating that eggs are 1.5 times more likely to hatch in the rainy season than in the dry season.

**Table 1:** Abundance of mosquito eggs collected during dry and rainy periods in the SANA Environmental Protection Area, Macaé, Rio de Janeiro, Brazil, from 2016 to 2018.

Eggs	Dry period	Rainy period	Total collected
Hatched eggs	1,074	10,055	11,129
Unhatched eggs	310	1980	2,290
Total collected	1,384	12,035	13,419

**Fig 2:**
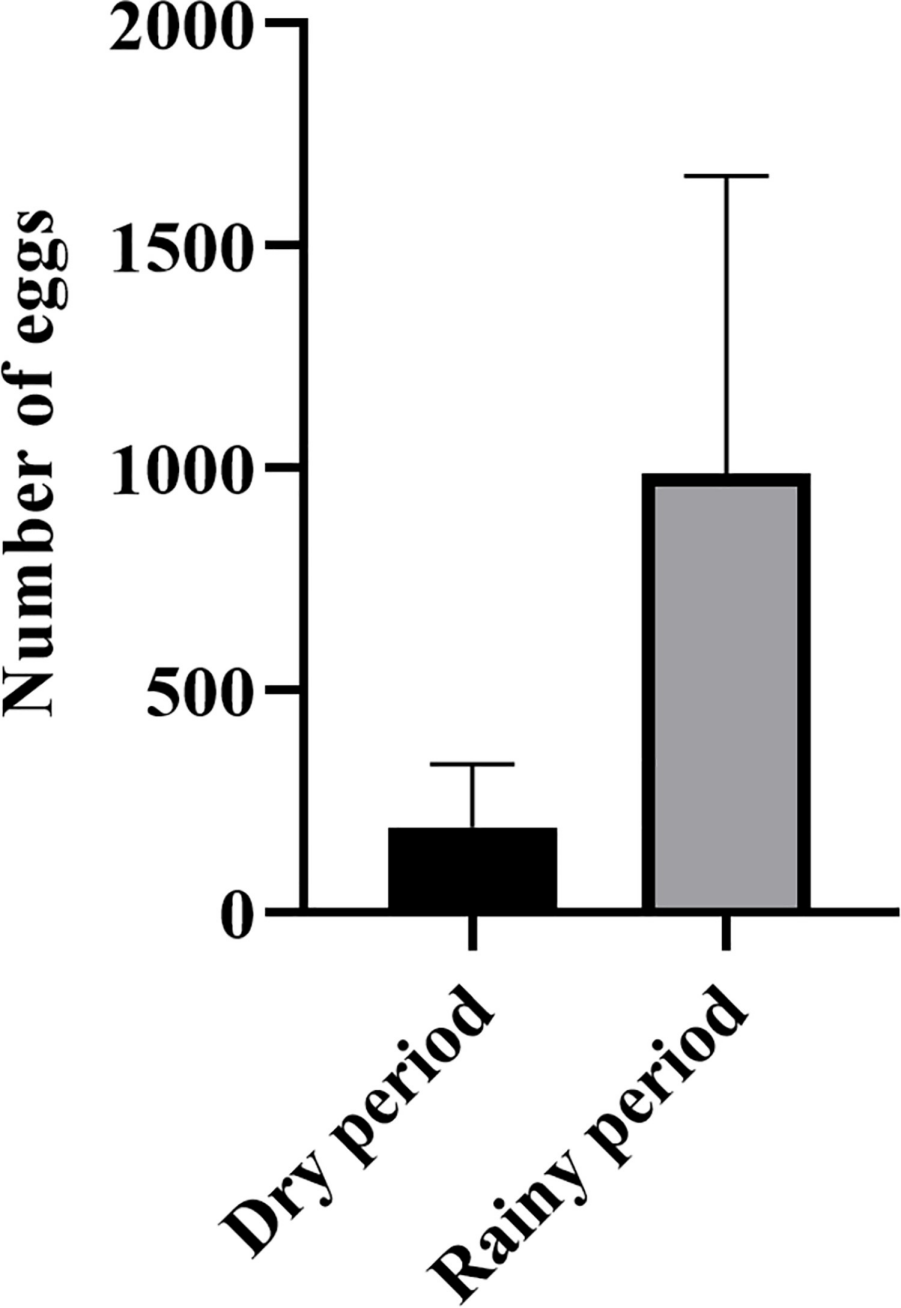
Comparative analysis of the *Hg*. *leucocelaenus* mean number of eggs (± S.D.) collected during the dry (Aug/18, Jun/19, Jul/19) and rainy (Dec/18, Jan/19 and Feb/19) periods in the SANA Environmental Protection Area, Macaé, Rio de Janeiro, Brazil, from 2016 to 2018.

**Fig 3:**
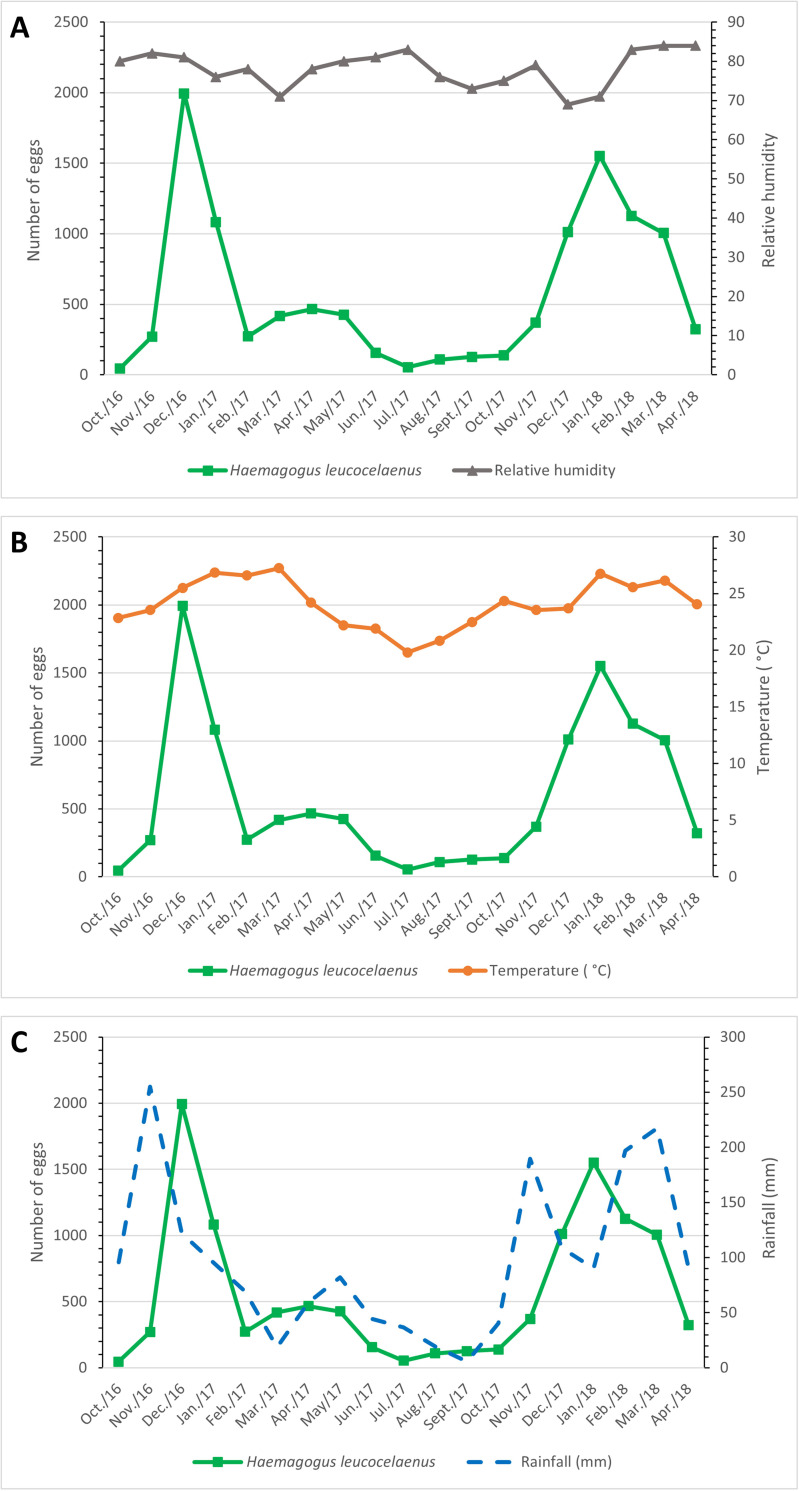
Variation in the number of *Hg*. *leucocelaenus* eggs concerning (A) relative humidity, (B) temperature, and (C) precipitation recorded in the SANA Environmental Protection Area, Macaé, Rio de Janeiro, Brazil, from 2016 to 2018.

The monthly average (± S.D.) of *Hg*. *leucocelaenus* eggs collected in all ovitraps was 193.1 (±141.1) in the dry season and 989.4 (±667.9) in the rainy season. These values are significantly different according to Welch’s t-test (t = 3.980, df = 12.610, p = 0.0017) (Fig 2). The abundance of *Hg*. *leucocelaenus* eggs was significantly and positively correlated with relative air humidity (r = 0.507; p< 0.027) (Fig 3A) and temperature (r = 0.670; p = 0.002) (Fig 3B). On the other hand, we found no significant correlation between egg abundance and precipitation (r = -0.068; p> 0.0783) (Fig 3C).

Each of the 15 installed ovitraps captured, on average (±S.D.), 892 (±1,002) eggs of *Hg*. *leucocelaenus* during the study period. The values of OPI and EDI (CI 95%) were 80.5% (74.5–86.5) and 36.6 (22.8–49.9), respectively. In the dry and rainy seasons, the OPI values (CI 95%) were 69.3% (61.5–77.2) and 91.7% (87.0–96.3), respectively. The EDI values (CI 95%) were 14.3 (10.2–18.4) and 58.4 (36.0–80.8), respectively.

## Discussion

There has been a growing interest in studying the biology and ecology of *Hg*. *leucocelaenus* populations due to their role in the wild cycle of YFV and, potentially, other arboviruses [29]. The species is abundant in the state of Rio de Janeiro, where the circulation of YFV in wild environments has been widely recorded [30]. In the present study, *Hg*. *leucocelaenus* was the dominant species in the ovitraps installed. Along with the other two species collected (*Ae*. *terrens* and *Hg*. *janthinomys*), *Hg*. *leucocelaenus* has been found to reproduce naturally in the breeding areas formed by tree holes [3, 31].

The hatching rates of mosquito eggs showed marked differences between the two periods analyzed, with eggs from the rainy period 1.5 times more likely to hatch than those from the dry period. This pattern in hatching rates between dry and rainy periods probably reflects what occurs for *Hg*. *leucocelaenus* in the nature since this species represented almost all individuals identified in the present study.

Although the species’ eggs have a certain tolerance to desiccation [32], their viability may be negatively affected in drier periods when the amount of rain is insufficient to keep the breeding site moist or when it may not allow the water level to reach the eggs repeatedly. The resistance level to dissection varies among mosquito species. The period of up to three hours after oviposition, at the beginning of embryogenesis and before the formation of the waxy cuticle, is when the egg is most susceptible to lack of moisture. Furthermore, even after the formation of a waxy cuticle, which prevents water loss from the interior of the egg to the external environment, some species remain highly susceptible to desiccation in the breeding site, as is the case of *Culex* species [33]. In addition to the moisture of the breeding site, the number of immersions that *Hg*. *leucocelaenus* eggs undergo during embryogenesis seems to be an important factor in hatching rates [7]. Thus, rainy and hot periods offer greater possibilities for multiple immersions of eggs. According to Evangelista et al. [34], the combination of high temperatures and precipitation recorded during the rainy summer favors YFV transmission and geographic dissemination of epizootic waves due to the positive influence of the increased hatching rates and the acceleration of larval development.

In many natural breeding sites formed by tree holes, their aquatic content may be less exposed to climatic variations than the content of the ovitraps used in the present study, which have a wide opening in their upper part. Some tree holes may have small openings to the external environment that could limit water loss water through evaporation and reduce temperature variations in their interior [35, 36]. In addition, many breeding sites formed by tree holes have their aquatic content maintained by the plant’s physiological water [37], difficulting desiccation even in drier periods. Therefore, the differences in hatching rates of mosquito eggs observed between the dry and rainy season in the present study could have been overestimated by the use of traps. Instead, such differences could have been associated with variations in the water level in the breeding sites or with variations in humidity at particular stages in the embryogenesis of the eggs.

The present study demonstrated that *Hg*. *leucocelaenus* produces more eggs during the rainy season, which is similar to the observations made by Alencar et al. [38], who verified population spikes in *Hg*. *leucocelaenus* during the rainy season in a study in the state of Rio de Janeiro carried out in the Ecological Reserve of Guapiaçu. In a study in the Amazon, [39] found higher abundance and richness of mosquitoes in the rainy season compared to the dry season, with *Hg*. *leucocelaenus* only present during the rainy season. These findings are also in line with [40, 41], who note that *Haemagogus* species are generally captured in the wettest and hottest months.

The abundance of eggs identified in the ovitraps was positively correlated with climatic factors such as temperature and relative humidity but not with precipitation. Although it is known that climatic factors influence the abundance of vector mosquitoes and, consequently, affect the dynamics of vector-borne diseases, it is not always easy to establish this relationship [10]. Factors associated with environmental characteristics such as the type and density of vegetation cover, as well as the abundance of hosts and available breeding sites, can overcome the effect of climate on mosquito populations [42]. Other studies on the relationship between the abundance of immature or adult forms of *Hg*. *leucocelaenus* and climatic variables are controversial [6, 9, 42], reinforcing that the variations in this species have multifactorial causes. In addition, the fact that we purposefully added 300 ml of water to each ovitrap may have influenced our findings, especially in the absence of correlation between the abundance of *Hg*. *leucocelaenus* and the accumulated precipitation of the month. In drier months, such as October 2016 and July 2017, no ovitraps were found to be completely devoid of water.

In the present study, we show that ovitraps are effective for collecting *Hg*. *leucocelaenus* eggs in a wild environment. The ovitraps simulate breeding sites formed by tree holes, which retain rainwater, enabling the development of the mosquito fauna [31]. Over the 19 sampling months, each ovitrap captured an average of approximately 900 eggs. This figure was reflected in the high OPI (80.5%) and EDI (36.6) values, especially in the rainy season, where OPI and EDI values were 91.7% and 58.4, respectively. This may indicate that ovitraps can be used to capture and monitor *Hg*. *leucocelaenus* populations in wild environments, similarly to *Ae*. *aegypti* in urban areas [43]. Because they are easy to install and can be evenly distributed in the environment, ovitraps are a method that offers standardized sampling and is particularly useful in monitoring *Hg*. *leucocelaenus* in areas with YFV circulation. This finding was previously supported by Alencar et al. [42], who observed that ovitraps were able to collect *Hg*. *leucocelaenus* eggs at different heights in the forest and seasons.

Monitoring YFV vector populations is important since there is a degree of periodicity in disease outbreaks, which occur about 1.4 to 2.7 years apart, depending on the type of environment [24]. We found that ovitraps can be effective for monitoring *Hg*. *leucocelaenus* populations in the study region, especially in the rainy season. In addition, our data suggest that this season is more important than the dry season for the production and probably hatching of *Hg*. *leucocelaenus* eggs in the study region, which can be considered in the elaboration of surveillance programs of YFV.

## Supporting information

S1 Fig Photo of the ovitrap installed in the forest to collect mosquito eggs. (TIF)

S1 Table Data from mosquito collections, carried out in Sana Environmental Protection Area (SPA), municipality of Macaé, State of Rio de Janeiro, Brazil. (XLS)
