# Supplementary figures and images for: Oviposition activity of Haemagogus leucocelaenus (Diptera: Culicidae) during the rainy and dry seasons, in areas with yellow fever virus circulation in the Atlantic Forest, Rio de Janeiro, Brazil
Source: PLoS One. 2021 Dec 13;16(12):e0261283. doi: 10.1371/journal.pone.0261283 (PMC8668088; doi:10.1371/journal.pone.0261283)

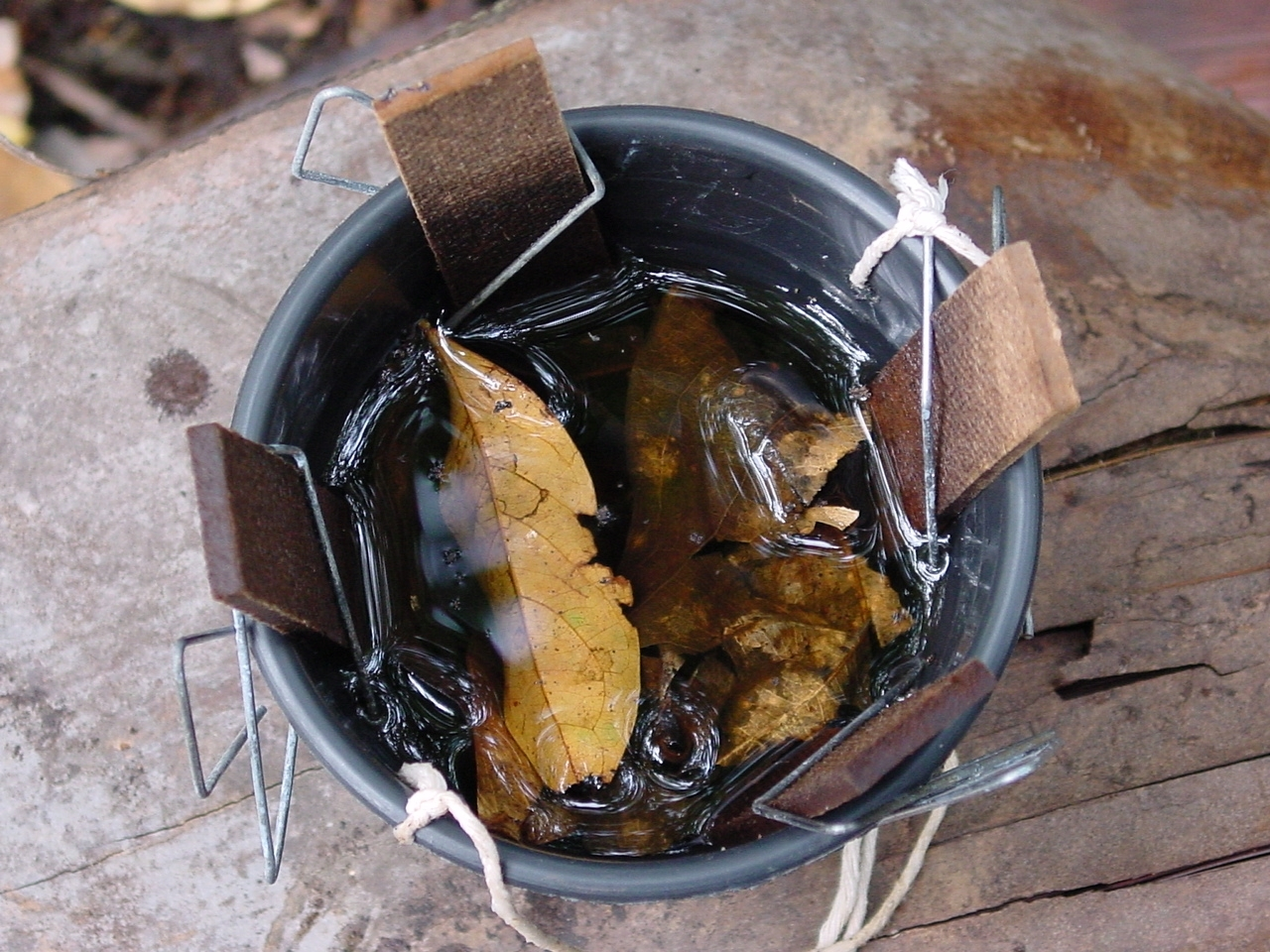

Supplement: S1 Fig — (TIF) [file pone.0261283.s001.tif]
